# From Waste to Value: Extraction of Protease Enzymes from Brewer’s Spent Yeast

**DOI:** 10.3390/foods14030503

**Published:** 2025-02-05

**Authors:** Marie Schottroff, Klara-Marie Jaeger, Ana Malvis Romero, Mark Schneeberger, Andreas Liese

**Affiliations:** 1 Institute of Technical Biocatalysis, Hamburg University of Technology, Denickestrasse 15, 21073 Hamburg, Germanyana.malvis.romero@tuhh.de (A.M.R.); 2 United Nations University Hub on Engineering to Face Climate Change at the Hamburg University of Technology, United Nations University Institute for Water, Environment and Health (UNU-INWEH), Am 8 Schwarzenberg-Campus 3, 21073 Hamburg, Germany; 3 GEA Brewery Systems GmbH, Heinrich-Huppmann-Strasse 1, 97318 Kitzingen, Germany; mark.schneeberger@gea.com

**Keywords:** Brewer’s spent yeast, homogenization, autolysis, sustainable valorization, protease, enzyme extraction

## Abstract

This study investigates the potential of additive-free extraction techniques to produce a proteolytically active yeast extract for use in the food industry. Brewer’s spent yeast, a by-product of the brewing industry, is utilized as a feedstock, and thus a new route for its valorization is proposed. Four methods of releasing these components while maintaining their intrinsic bioactivity are investigated: thermal autolysis, ultrasonication, cell milling and high-pressure homogenization. Thermal yeast autolysis resulted in the highest release of protease activity, with 2.45 ± 0.05 U/g_dm_ after 3 h incubation at 45 °C. However, autolysis poses challenges for automation, and thus a stop criterion, due to the lack of in-line enzyme activity assays,. While glass bead treatment gave the highest reproducibility, ultrasonication and high-pressure homogenization resulted in comparably high protease activities in the BSY extracts produced. Both methods, in the form of a cell mill and high-pressure homogenizer, are cell disruption methods that are already employed on an industrial scale. It has now been demonstrated that these methods can be used to produce proteolytically active yeast extracts from a previously considered waste stream.

## 1. Introduction

Brewer’s spent yeast (BSY) is the second most abundant by-product of the brewing industry, accounting for approximately 1.5–2.5% of the total beer produced [1]. The cells are still viable and considered food grade when they leave the process. However, to date, they are mostly sold as low-cost animal feed or disposed of in landfills, which is not in line with the UN sustainable development goals (SDG) [2]. Valorizing BSY into valuable products can support SDG 2 “Zero Hunger”, in the case of food-grade applications; SDG 9 “Industry Innovation and Infrastructure”, by enabling new processing routes for this by-product and SDG 12 “Responsible Consumption and Production”, by introducing a valorization process where currently a waste stream is produced. Moving towards these goals together with the global availability of BSY has led to extensive research into the valorization of this by-product [3]. Recent approaches have focused on the use of BSY as a fermentation substrate and the extraction of valuable fractions such as proteins, peptides and glucans as functional foods [3,4]. The direct use of its nutritional value is linked to the amino acid profiles of BSY and its extracts with compositions attractive for human consumption [2]. In addition, peptide-rich extracts of BSY have been associated with multiple bioactivities, including antimicrobial, antioxidant and antihypertensive properties [2,5]. Next to the extraction of bioactive peptides from BSY, intrinsic bioactivities from enzymes of the yeast cells offer a different potential for valorization. Of particular interest are yeast-derived enzymes, most promisingly protease and invertase enzymes [5]. As proteases constitute the largest category within the industrial enzyme markets, the demand for novel, cost-effective and food-grade enzymes is high [6,7]. These bioactive molecules could potentially act as green hydrolysis feedstocks for use in the food and health sectors, where the production of peptide-rich products from (plant) protein sources is a highly sought after area of research [8,9,10]. However, there has been little research into the extraction of intracellular enzymes from BSY [11,12,13]. Most existing studies focus on the extraction of specific enzymes for analytical investigation of enzyme type and properties to gain knowledge on the yeast strain and its metabolism [14,15,16,17]. The focus on extraction of enzymes in a crude yeast extract to make use of their activity in subsequent processes is not well established.

BSY contained proteases are typically found in the vacuole compartment, an organelle responsible for degradation of excess proteins and storage of amino acids, among other functions [18]. In order to fulfill this function, contained proteases must have a wide substrate spectrum that could be biotechnologically used on protein sources relevant to the food sector.

The release of proteases into the extracellular medium requires an effective cell wall disruption, while limiting the degradation rate and loss of activity of the components of interest. Commonly used cell wall disruption processes for yeast include physical, chemical, enzymatic and mechanical methods. Physical methods include the application of decompression and osmotic shock; however, they are typically limited to lab-scale only and thus pose challenges for the development of industrially relevant processes [19]. Chemical methods comprise, among others, the use of acidic and alkaline solutions. The extraction of β-glucans from yeast has been proposed to be effective when using sodium hydroxide in concentration ranges of 0.5–1 M for 1–4 h [20,21]. However, loss of bioactivity can be assumed for the extraction of enzymes due to conformational changes linked to pH shifts [22,23]. These methods are thus limited for use in extracting enzymes from BSY with a high bioactivity.

Enzymatic cell wall disruption is either done through the addition of external enzymes, such as proteases, or via autolysis, a stress-induced self-degradation of yeast. Thermal yeast autolysis coupled with dosing of sodium chloride, ethyl acetate or an osmotic shock are industrially applied processes for yeast peptide and amino acid extracts [24]. In this case, bioactivity is mostly linked to the composition of peptides in the final product, rather than to functional protein molecules with enzymatic activity. Without the addition of external additives and pH shifts, this technology might still be of interest if stopped before thermal degradation or self-digestion of the released protease enzymes. The fourth major group of cell wall disruption methods includes mechanical treatments such as cell milling, typically with glass or zirconia beads, ultrasonication and microwave treatment [19]. This last group is typically applied when yeast extracts with larger bioactive molecules, such as enzymes, are the products of interest [19,25,26]. Van Gaver and Huyghebaert were able to e.g., extract two different cell wall bound enzymes when using a pilot-scale cell mill type CoBall-Mill MS-12 from FRYMA-MASCHINEN (Rheinfelden, Switzerland) [27].

Given the dependance of applied cell disruption methods on compositional profile and bioactivity in the resulting yeast extract, this study aims to identify suitable methods and operating conditions for a production of yeast extracts with maximum protease activity. The focus is set on the industrially relevant cell wall disruption methods thermal autolysis, cell milling, ultrasonication and high-pressure homogenization, enabling a fast application on larger scales. A valorization process is proposed using the food-grade brewing industry by-product brewer’s spent yeast as a green hydrolysis feedstock.

## 2. Materials and Methods

**Figure 1:**
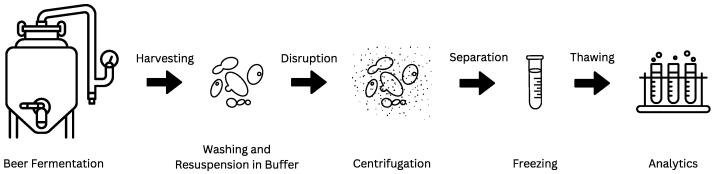
Schematic overview of the experimental procedure from beer fermentation to proteolytically active BSY extracts. Icons from © amethyststudio, Icons8, nessign, sparklestroke, effort_project and Victoruler via Canva.com.

Unless otherwise stated, all chemicals were purchased from Carl Roth (Karlsruhe, Germany) or Sigma Aldrich (Steinheim, Germany) in a purity ≥ 98%. The SafLager^TM^ W-34/70 yeast strain used, a *Saccharomyces pastorianus* variant, was purchased from Fermentis by Lesaffre (Marcq-en-Baroeul Cedex, France). The gel used for electrophoresis of proteins ServaGel^TM^ TG PRiME^TM^ 8% was purchased from SERVA (Heidelberg, Germany). Brewer’s spent yeast was produced in a 1 hL microbrewery before being stored for a maximum of 24 h at 4 °C prior to cell disruption. The experimental procedure from beer fermentation to determining the protease activity in produced BSY extracts is given in Figure 1. Details for each procedure are specified in the following sections.

### 2.1. Fermentation of Brewer’s Spent Yeast

A standardized American lager beer was brewed in the 1 hL microbrewery Campus Perle of the Hamburg University of Technology (Hamburg, Germany). The gravity after boiling was set to 12.6 °P. The bottom-fermenting lager yeast strain *S. patorianus* type SafLager^®^ W34/70 by Fermentis was used for all fermentations. Fermentations were run in 220 L Speidel FD-DS tanks (Ofterdingen, Germany) at 12.8 °C until a constant residual sugar content was determined for two consecutive days. The beer suspension was subsequently cooled to 7 °C for 24 h to allow the yeast to sediment in the cylindroconical bottom of the tank. The BSY slurry was harvested after beer separation and stored at 4 °C for a maximum of 24 h before cell disruption.

### 2.2. Washing of Brewer’s Spent Yeast Slurry

The BSY slurry was centrifuged in a Beckman Coulter Avanti J25 (Brea, CA, USA) at 3019 g at 4 °C for 10 min to separate beer from the yeast. The cell pellet was resuspended in ultrapure water and centrifuged again under the same conditions. The washing process was repeated a total of two times before resuspending the cell pellet at a ratio of 1:2 w/v in 0.1 M sodium phosphate citrate buffer at either pH 6. This suspension was used for all cell disruptions.

### 2.3. Thermal Yeast Autolysis

For the thermal yeast autolysis, 20 mL of the BSY suspension were transferred to 100 mL baffled Erlenmeyer flasks and placed in a preheated Infors HT ecotron incubator (Bottmingen, Switzerland) at a temperature of 45, 50 or 55 °C, respectively. Shaking was set to 200 rpm. The autolysis samples were left for a maximum of 4 h. At each sampling time, one flask was removed, and the content was centrifuged at 3857 g and 4 °C for 20 min in a Beckman coulter J2-HS centrifuge (Brea, CA, USA). The liquid supernatant was carefully transferred to a fresh test tube. The remaining cell wall debris was dried to constant weight.

### 2.4. Glass Bead Cell Disruption

Glass beads of 0.5 mm diameter were added at a ratio of 1:1 w/w cell pellet to glass beads to 20 mL of the buffered yeast suspension. The mixture was vortexed at 100% power input on a Scientific industries vortex genie (Bohemia, NY, USA) for the desired cell disruption time. Every 5 min, the mixture was cooled in an ice water bath for 5 min. BSY extract was separated from the cell wall debris via centrifugation in a Beckman coulter J2-HS centrifuge (Brea, CA, USA) at 3857× *g* and 4 °C for 20 min. The liquid supernatant was carefully transferred to a fresh test tube. The remaining cell wall debris was dried to constant weight.

### 2.5. Ultrasound Treatment

20 mL of the buffered yeast suspension were transferred to a 50 mL reaction vessel. A 20 kHz Bandelin MS 73 sonotrode (Berlin, Germany) was placed in the middle of the suspension. The reaction vessel was placed in an ice water bath before starting the ultrasound treatment at 100% power input for the desired cell disruption time. Every 2 min, the temperature of the suspension was checked. If a threshold of 15 °C was surpassed, the suspension was kept on ice for 5 min before resuming the cell disruption treatment. BSY extract was separated from the cell wall debris via centrifugation at 3857× *g* and 4 °C for 20 min in a Beckman coulter J2-HS centrifuge (Brea, CA, USA). The liquid supernatant was carefully transferred to a fresh test tube. The remaining cell wall debris was dried to a constant weight.

### 2.6. High-Pressure Homogenization

High-pressure homogenization was performed in a GEA PandaPlus NS 1001L homogenizer (Parma, Italy). For each run, 300 mL of buffered BSY suspension was disrupted for up to 10 passes through the high-pressure valve. The homogenized suspension was pumped back to the feeder after each pass through the valve. A countercurrent tubular heat exchanger with water as the cooling medium was used to maintain the temperature below 20 °C after each pass. The BSY extract was separated from the cell wall debris by centrifugation at 3857× *g* and 4 °C for 20 min in a Beckman coulter J2-HS centrifuge (Brea, CA, USA). The liquid supernatant was carefully transferred to a fresh test tube. The remaining cell wall debris was dried to constant weight.

### 2.7. pH Screening

A pH screening was performed to determine a suitable pH range for cell disruption. All cell disruptions were performed following the above procedure of glass bead treatment for 15 min. The buffers used during cell disruption are summarized in Table 1.

### 2.8. Determination of Yeast Vitality

Yeast vitality was assayed using an acidification power test adapted from Gabriel et al. [28]. The yeast vitality is defined as the ability to both metabolize endogenous glycogen and exogenous glucose substrates. The magnitude of spontaneous acidification (AP_10_) through hydrolysis of internal glycogen bonds is measured over a period of 10 min with a starting pH of 6.3. For this, the harvested BSY-beer slurry is washed twice with ultrapure water at pH 6.3 and centrifuged at 1000 rpm for 1 min and 20 °C in a Beckman coulter J2-HS centrifuge (Brea, CA, USA). Of the resulting BSY cell pellet, 3× *g* is resuspended in 15 mL ultrapure water at pH 6.3. The change in pH of this suspension is recorded every minute for a total of 10 min using a Knick 766 pH meter (Berlin, Germany). The magnitude of glucose-induced acidification power (AP_20_) is also measured for a period of 10 min directly after the AP_10_ measurement, where 4.5 mL of a 50% glucose solution at pH 6.3 is added. Both values are defined as per Equations (1) and (2).

(1) $${AP}_{10}=6.3-pH(t=10\;min)$$

(2) $${AP}_{20}=6.3-pH(t=20\;min)$$

### 2.9. Determination of Image-Based Cell Disruption Efficiency

For the determination of an image-based cell disruption efficiency, a light microscopic analysis on a Nikon Eclipse H550L (Tokyo, Japan) at 40× magnification was conducted. Digital analysis was carried out using the Software Nikon NIS-Elements AR (Tokyo, Japan).

### 2.10. Determination of Proteolytic Activity

Quantification of enzyme activity was done via a non-specific protease assay as described by Cupp-Enyard et al. [29]. The activity was determined spectrophotometrically at 660 nm in triplicates with an extinction coefficient of 9.8505 L/mmol/cm. One unit is defined as the production of a color equivalent to 1 µmole of tyrosine per minute at pH 7.5 and 37 °C. The reaction was started by adding 1 mL of enzyme solution to vials containing 5 mL of a 0.65% by mass casein solution prepared in 50 mM potassium phosphate buffer pH 7.5 preheated to 37 °C. No enzyme solution was added to the blank. The reaction was stopped after 10 min by adding 5 mL of a 110 mM trichloroacetic acid solution. 1 mL of enzyme solution was then added to the blank. All vials were incubated for another 30 min at 37 °C before 2 mL were filtered over 0.45 µm polyether sulfone syringe filters into a suitable vial. Then, 5 mL of a 500 mM sodium carbonate and 1 mL of 0.5 M Folin’s phenol reagent were added. After another 30 min incubation at 37 °C, samples were filtered again, and 1 mL was transferred into suitable cuvettes for absorbance measurement at 660 nm. A standard curve was recorded with l-tyrosine. Activity was calculated according to Equations (3) and (4),

(3) $$v_{V}=\frac{n_{tyrosine}\cdot V_{assay}}{t{\cdot V}_{enzyme}\cdot V_{cuvette}}$$

(4) $$v_{S}=\frac{v_{V}}{c_{BSY,DM}}$$

where $v_{V}$ is the volumetric activity in U/mL, $n_{tyrosine}$ is the equivalent amount of tyrosine in the assay determined from the slope of the calibration curve in µmol, $V_{assay}$ is the assay volume in mL, t is the reaction time in min, $V_{enzyme}$ is the enzyme solution volume used in mL, $V_{cuvette}$ is the cuvette volume in mL, $v_{S}$ is the mass specific activity in U/g based on BSY dry matter and $c_{BSY,DM}$ is the concentration of BSY dry matter in the enzyme solution in g/mL.

### 2.11. Determination of Protein Content in BSY Extracts

A colorimetric assay was chosen to qualitatively assess the protein contents in the produced BSY extracts. The basis for the Pierce bicinchoninic acid is a reduction of Cu^2+^ to Cu^+^ by protein in an alkaline medium [30]. In short, 150 µL of Pierce reagent were mixed with 10 µL of sample, followed by 5 min incubation at 30 °C in the dark in a microwell plate. Absorbance was read at 660 nm in a Tecan Infinite 200 Pro microplate reader (Maennedorf, Switzerland). Calibration was done with bovine serum albumin (R^2^ = 0.9947). Since this assay was only an indirect assay, protein content quantification was done with an amino acid high-performance liquid chromatography (HPLC).

### 2.12. Determination of Amino Acid Profile and Protein Extraction Yield

The amino acid profiles of BSY samples were determined according to Lamp et al. [31]. In short, quantification of proteinogenic amino acids was carried out chromatographically after analytical acid hydrolysis. Samples were hydrolyzed at 110 °C for 24 h with 6 M hydrochloric acid (HCl) in a convective oven. After cooling, the sample pH was adjusted to pH 1 using 10 M sodium hydroxide (NaOH), and 2 mL of internal standards L-Norvaline and Sarcosine, each at 2.5 mM, were added and mixed. Samples were filtered through a 0.45 µm polyether sulfone syringe filter and transferred to HPLC vials. For chromatographic analysis, an Agilent Infinity 1260 HPLC (Santa Clara, USA) with fluorescence detector was used, where the nonpolar stationary phase is a C18 column and the mobile phase is a gradient system of an aqueous buffer solution of 0.5 M sodium borate buffer at pH 8.4 and an organic phase of acetonitrile, methanol and water at a volumetric ratio of 45:45:10. Derivatization was done with o-phthaldialdehyde (OPA) for aspartic acid, glutamic acid, serine, histidine, glycine, threonine, arginine, alanine, tyrosine, methionine, phenylalanine, isoleucine, leucine and lysine and with 9-fluorenylmethyloxycarbonyl chloride (FMOC-Cl) for proline. The protein extraction yield was based on the determined amino acid profile and defined according to Equation (5),

(5) $$Y_{P}=\frac{m_{H}\cdot w_{P,H}}{m_{BSY}\cdot w_{P,BSY}}$$

with $Y_{P}$ denoting the extraction yield, $m_{H}$ the dried mass of the hydrolysate in g, $w_{P,H}$ the protein content in the hydrolysate per dry matter hydrolysate in g/g, $m_{BSY}$ the dry matter mass of BSY in g and $w_{P,BSY}$ the protein content in BSY per dry matter of BSY in g/g. The protein content of the hydrolysate could not be determined directly due to limits in available sample mass after drying. It was therefore calculated via a mass balance as given in Equation (6) based on the determined protein content in the solid residue after drying,

(6) $$m_{H}w_{P,H}={1-m}_{SR}\cdot w_{P,SR}$$

with $m_{SR}$ denoting the dried mass of the solid residue in g and $w_{P,SR}$ the protein content in the solid residue per dry matter in g/g.

### 2.13. Determination of Protein Sizes

Protein sizes were evaluated qualitatively via a sodium dodecyl sulfate-polyacrylamide gel electrophoresis (SDS-Page). The polyacrylamide concentration in the gels was 8%. The protein marker used visualized protein sizes between 10–245 kDa. For sample preparation, 20 µL of sample were mixed with 10 µL of ultrapure water and 10 µL of SDS buffer solution. Sample proteins were then denatured at 80 °C for 5 min. 10 µL of the samples were pipetted into the gel chambers submerged in the SDS running buffer consisting of 30 g/L tris-(hydroxymethyl)-aminomethan, 144 g/L glycine and 10 g/L SDS. The voltage was set to 170 V for 30–60 min. The gel was then washed with ultrapure water and stained for 1 h with Coomassie blue under constant shaking.

### 2.14. Statistical Analysis

The given data represents averages derived from three independent experiments or measurements. An exception is the highpressure homogenization trials, where only one technical replicate was conducted due to limits in the available BSY mass. The results are expressed as average ± standard deviation. A paired two-tailed t-test was used to detect significant differences between two individual cell disruption methods. The significance level is either 0.05 or 0.01, designated by lower-case and capital letters, respectively, and is always reported. In addition, a one-way analysis of variance (ANOVA) was performed to identify significant differences between all four cell disruption methods.

## 3. Results and Discussion

### 3.1. pH Screening

**Figure 2:**
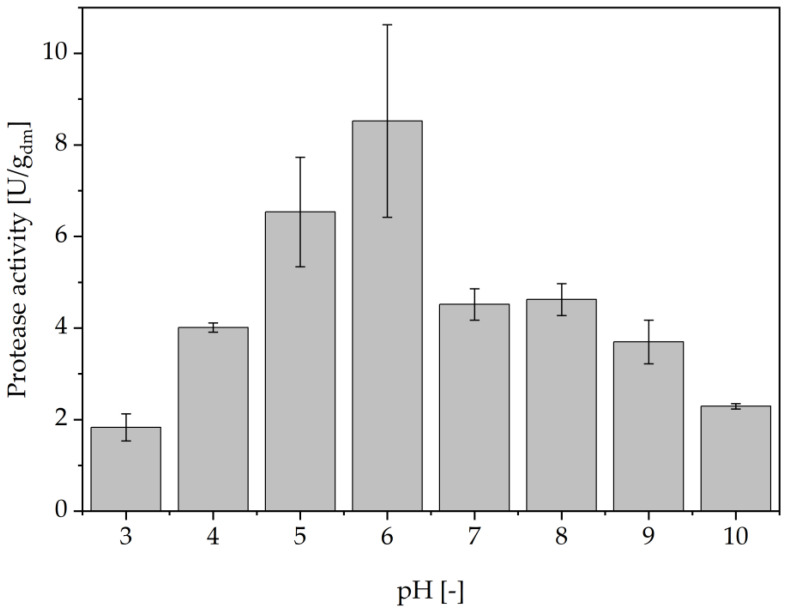
Protease activity in BSY extracts at different pH. All buffers used at 0.1 M concentration. pH 3–7 with Na_2_HPO_4_ + C_6_H_8_O_7_ buffer, pH 8–9 with C_4_H_11_NO_3_ + HCl buffer and pH 10 with Na_2_CO_3_ + NaHCO_3_ buffer.

Prior to evaluating the influence of different cell disruption methods, a pH screening was conducted to identify a suitable pH range in which differences in proteolytic activity are expected to be readily apparent. Figure 2 summarizes the proteolytic activities determined after cell disruption with 0.5 mm glass beads for 15 min at 4 °C. All investigated scenarios resulted in proteolytic activity in the cell-free supernatant. The highest overall activities were found at pH 5 and 6 in 0.1 M sodium phosphate citrate buffer. These pH values correspond to the commonly reported intracellular pH in *S. cerevisiae* as well as in the vacuole compartment itself [32,33]. Therefore, most of the seven known vacuolar proteases are expected to be in their active conformation at these pH values [34]. This assumption is further supported by the found activity at acidic conditions at pH 3–4, which may be related to protease A, an important exoprotease for the activation of other vacuolar proteases [15,34,35]. Next to the maximum activity found at pH 6, Maddox et al. could demonstrate maximum stability of different vacuolar proteases extracted from *S. carlsbergensis*, a common beer yeast strain, at pH values of 6–6.5 [15]. In accordance with these findings and the presented data, pH 6 was chosen for subsequent studies of the different cell disruption methods.

### 3.2. Thermal Yeast Autolysis

**Figure 3:**
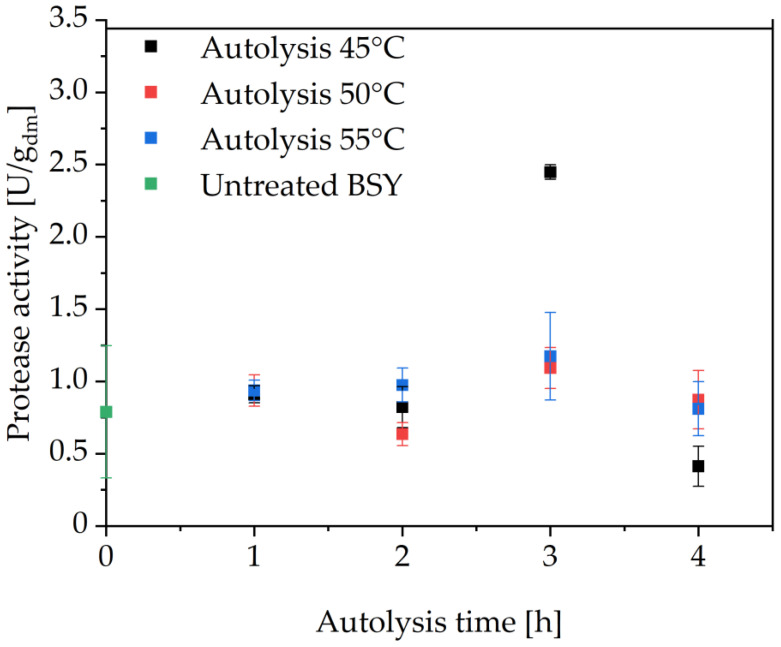
Protease activity in thermally autolyzed BSY extracts at pH 6 in 0.1 M sodium phosphate citrate buffer. No significant differences between autolysis runs at 50 °C and 55 °C in a two-tailed t-test at a significance level of 0.05. All other combinations show significant differences at the same significance level. BSY: brewer’s spent yeast.

An industrially relevant technology for the production of commercial yeast extracts is thermal yeast autolysis [25,36]. This stress-induced self-degradation of yeast cells by endogenous enzymes can be triggered by external factors such as temperatures above 45 °C, pH shock, addition of salts or ethyl acetate [24,37]. The acting endogenous enzymes are mostly linked to proteases and carbohydrases, such as β-glucanases [24]. For brewing yeast strains, thermal yeast autolysis has shown to result in up to 98% cell disruption effectiveness through lysis of the cell wall by intracellular enzymes [25,38]. To test, if it is possible to release intracellular proteases and produce proteolytically active yeast extracts via this technology, a set of screening experiments was conducted for 24 h (Supplementary Materials Figure S1). The data were inconclusive regarding trends of maximum released proteolytic activity with time and temperature; however, it revealed that, above 4 h of incubation, there was no further increase in activity. As thermal inactivation of yeast extracted proteases has been reported to occur at temperatures above 45 °C, it is assumed that this is the major reason for no further increase in the determined protease activity [15]. It is possible that, after 4 h, the thermal inactivation and self-degradation of released proteases are equal to or higher than the protease release rates through the progressing autolysis. The highest overall proteolytic activity in the present study was determined at 45 °C after 3 h incubation with a value of 2.45 ± 0.05 U/g_dm_, as can be seen in Figure 3, with a drastic decrease to 0.41 ± 0.14 U/g_dm_ at the same temperature after 4 h of incubation. Thermal inactivation of the protease enzymes is assumed to be the predominant factor, as brewing yeast strains are typically selected for optimal growth in the 12–20 °C range [39]. In a previous study by Woods and Kinsella, it was reported that extracted proteases from a strain of *S. carlsbergensis* showed no thermal inactivation at temperatures in the range of 25–37 °C over the course of 40 min, whereas complete inactivation occurred after 5 min at 70 °C and pH 6 [14]. Maddox and Hough also reported thermal inactivation of proteases extracted from *S. carlsbergensis* at temperatures above 50 °C after 1 h incubation at pH 6.3–6.6 [15]. As the present work examined a crude yeast extract containing several different enzymes and not isolated fractions as in the aforementioned studies, it is assumed that not all proteases are thermally inactivated at the same time and rate. However, it is hypothesized that the majority of extracted proteases are inactivated between 3–4 h of autolysis. This highlights the importance of stopping the autolysis process at the time of maximum protease activity. However, to date, there is no inline protease activity measurement available, posing challenges in process automation and raising the question of the overall feasibility of this processing approach for producing proteolytically active yeast extracts.

**Figure 4:**
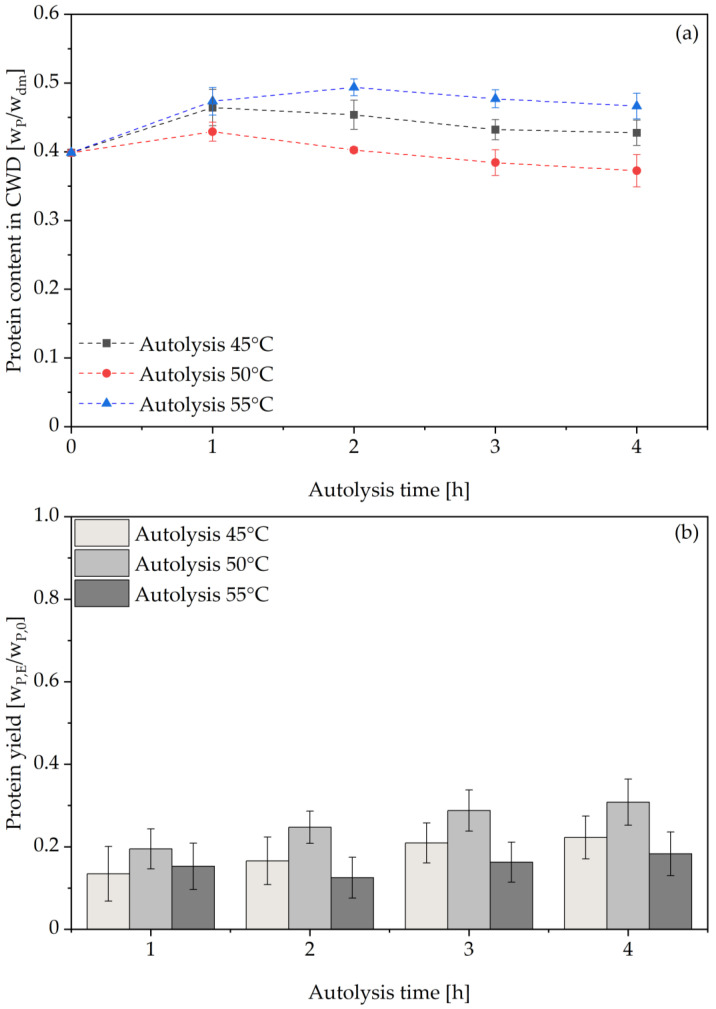
Protein content (**a**) in residual cell wall debris fraction of thermally autolyzed yeast cells at pH 6 in 0.1 M sodium phosphate citrate buffer. Protein extraction yield (**b**) for BSY extract calculated via mass balance. Significant differences between autolysis run at 45 °C and 50 °C for protein content and between 45 °C and 55 °C for protein yield in a two-tailed t-test at a significance level of 0.01. All other combinations show no significant differences. CWD: cell wall debris.

Next to the overall released protease activity, protein extraction efficiency is an important parameter commonly used to compare different cell disruption methods [25]. However, due to limits in dry mass of the produced liquid hydrolysate fractions, the protein content of the residual cell wall debris was determined as shown in Figure 4a. As can be seen, the dry mass specific protein content increases above the protein content of untreated BSY cells for all investigated autolysis runs, before decreasing again. This is assumed to be due to a dry matter loss of the BSY cells due to the progressing autolysis. At longer autolysis times, this protein content decreases again, as released proteases from the yeast cells also degrade insoluble protein from within the cells to soluble peptides and amino acids released to the extracellular medium. To account for this dry matter loss, a comparison of protein extraction yields of the BSY extracts was calculated via a mass balance as described in the Section 2.12. This also enables a comparison of the obtained results with other studies, as the mass-specific protein content can vary depending on the metabolic state of the yeast at the point of harvest and also between different yeast strains applied. When evaluating the overall protein extraction efficiency, a trend of increasing protein yield in the BSY extract over time can be seen in Figure 4b. Of the temperatures tested, the autolysis run at 50 °C resulted in the highest total protein extraction yield of 32.3 ± 3.6% by mass after 4 h of incubation. These results are consistent with previous studies by other groups that also found the maximum protein yield at 50 °C for BSY [25,40]. However, protein extraction yields were evaluated at later time points, as only protein extraction was of interest and not any residual protease activity in the extracts produced. The reported yields are in the range of 10–50% after 6–8 h of incubation. However, it is difficult to compare absolute values because most working groups do not report a measure of yeast vitality prior to cell disruption. This would be necessary to assess the metabolic state of the yeast cells prior to the start of autolysis and could explain different times for achieving high protein extraction yields. In addition, the exact yeast strain used is not consistently reported, nor are the beer fermentation and pretreatment conditions applied. However, both parameters are linked with the starting protein content of the yeast prior to cell disruption and thus required for a comprehensive comparison among studies. Jacob et al. also added inducers to the thermal yeast autolysis process, such as 0.086 mol/L sodium chloride and 0.051 mol/L ethyl acetate [25]. Another important factor to consider is the protein content determination method. In the present study, amino acid HPLC was used for all reported total protein contents. In the above-mentioned studies, indirect measurements such as Kjeldahl nitrogen determinations were used for this means, making direct comparisons of the protein yields obtained difficult.

**Figure 5:**
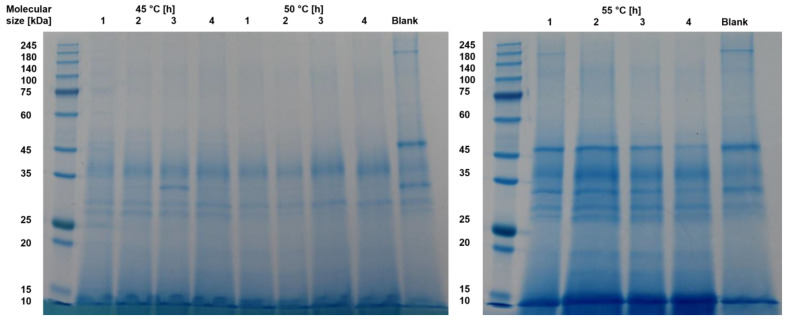
SDS-Page results for thermally autolyzed BSY extract samples.

Residual protease activity in BSY extracts requires the presence of intact proteins. To qualitatively assess the presence of differently sized proteins, an SDS-Page was performed. The resulting stained gels for the autolysis samples are shown in Figure 5. A darker blue color represents an increasing protein concentration. The blank refers to non-autolyzed cell-free supernatant from a BSY cell pellet suspended in 0.1 M sodium phosphate citrate buffer at pH 6.

The general trend for all temperatures examined shows that proteins larger than 45 kDa visible in the blank appear to be degraded over time to smaller proteins and peptides at all temperatures examined. This may be a result of either thermal degradation over time or the effect of protein hydrolysis by BSY proteases released during the autolysis process. Furthermore, this suggests that the autolysis process is not yet advanced enough to release cell wall-bound proteins, which are commonly found in the 60, 80, 145 and 220 kDa size ranges [41,42]. These would then have been separated as a pellet after centrifugation. This assumption is supported by the residual protein content of 40–45% found in the cell wall debris after 1 h of incubation. Another possibility is that these proteins are not released from the cell wall structure as a whole, but rather hydrolyzed in place and directly reduced to a peptide fraction by the action of proteases. For all investigated samples, the most prominent bands are visible for peptides with a size < 15 kDa. Other relevant bands are present in the range of 25–45 kDa. This could be attributed to the action of released vacuolar yeast proteases. In *S. cerevisiae* strains, there are seven known vacuolar proteases, which can be seen with their corresponding molecular sizes in Table 2.

Of the listed proteases, only two are endoproteases, namely proteinase A and B. Proteinase A plays an especially important role in the catalytic activation of other vacuolar proteases under stress conditions [34]. From the SDS-page results in this study, it can be seen that whenever a protein band at 30–35 kDa is visible, the color intensity of protein bands in the range up to 45 kDa increases over the course of the next hour. This might be linked to the release of proteinase A and subsequent activation of other vacuolar proteases, which subsequently hydrolyze yeast proteins to smaller peptides. The high color intensity at the lower end of the gels might be attributed to the action of vacuolar exopeptidases, which hydrolyze proteins to small peptides and free amino acids. Thermally induced yeast autolysis is known to result mostly in small peptides and amino acids, which is in line with the findings of the current study. Podpora et al. reported an increase in free amino acids during BSY autolysis from 11.2% after 2 h to 77.5% after 48 h at 47 °C and pH 5.2–6.2, linking it to decomposition of proteins and peptides [44]. In addition, Jacob et al. reported autolysis to yield the highest free amino acid content compared to cell disruption methods, cell mill and ultrasound sonotrode [25]. The main reason was hypothesized to be the predominant action of proteases during autolysis.

### 3.3. Glass Bead and Ultrasound Cell Disruption

**Figure 6:**
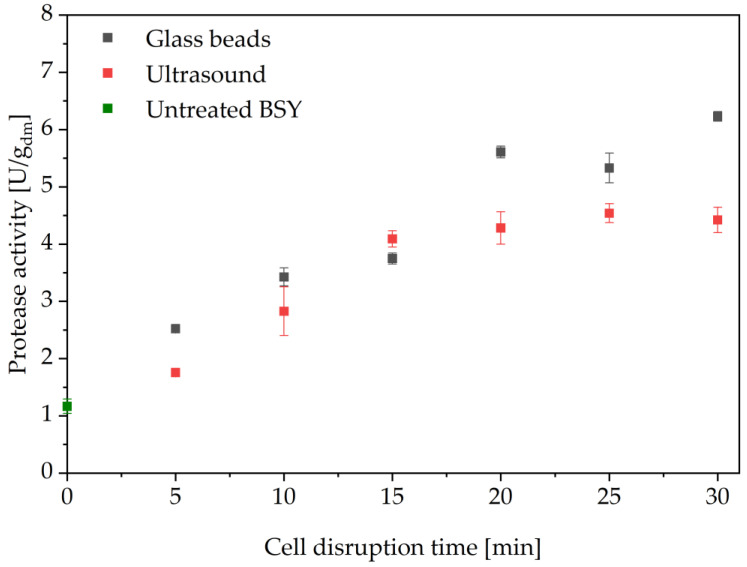
Protease activity in BSY extracts produced via 0.5 mm glass beads on a vortex mixer at 100% power input and 20 kHz ultrasound treatment. BSY suspended in 0.1 M sodium phosphate citrate buffer at pH 7. Differences between the two methods are significant at the 0.01 level using a two-tailed t-test. BSY: brewer’s spent yeast.

Mechanical cell disruption methods are an emerging technology for large-scale cell disruption where bioactivity is required in the extracts produced [19,45]. These processes do not require elevated temperatures for their cell wall disruption mechanism and typically run at times below 30 min. Glass bead and ultrasound treatments are two of the most common lab-scale cell disruption methods. Both are known to follow first order kinetics for protein release [46,47,48,49]. It has also been shown for BSY that after 15 min of cell disruption, first-order kinetics cease to apply, and instead the overall protein release follows an asymptotic form with only a small increase in released protein content, making it economically difficult to argue for longer treatment times [25]. In this study, a first set of screening experiments confirmed these findings for the release of protease activity for ultrasound treatment, as shown in Figure 6. After 15 min of this treatment, an asymptotical trend can be seen, reaching a maximum activity of 4.54 ± 0.17 U/g_dm_ after 25 min of cell disruption. Possible reasons for reaching a plateau of released protease activity might be related to the working principle of ultrasound. The cell disruption is a result of the cavitation of bubbles, which leads to strong local shear forces and high pressure as well as temperature gradients [50,51]. The combined effects result in the disruption of first the cell wall, followed by the cell membrane and thus a release of intracellular components. This was proposed by Zhang et al. after observing that cell wall-derived carbohydrates were predominantly released during the first 15 min of sonication of yeast cells before reaching a plateau at 20 kHz ultrasound frequency. Protein release reached a plateau after 20 min of ultrasound treatment, indicating that the degradation of first the cell wall and then the cell membrane had been fully achieved. [51] For this study, similar effects are assumed to be the cause of the asymptotic curve for protease activity release, where the plateau of maximum activity is reached after 20 min of ultrasound treatment. This could be related to the complete breakdown of both the cell wall and the cell membrane, including the membrane of the vacuole compartment. As the applied frequency of 20 kHz is at the lower end of ultrasound frequencies, it is assumed that the released proteases are not thermally or mechanically degraded during the 30 min treatment time, which could explain the constant protease activity in the time interval between 20 and 30 min [50]. However, it can also be assumed that longer treatment times can reduce the released protease activity due to prolonged local effects of high pressure and temperature gradients as well as the shear forces applied, leading to structural disintegration of the enzymes.

For the glass bead treatment, there is no clear asymptotic trend for the released protease activity. Increasing the cell disruption time beyond 15 min still resulted in increased protease activity and no clear plateau was reached. The highest overall protease activity was determined after 30 min cell disruption with 6.23 ± 0.09 U/g_dm_. As reported by van Gaver and Huyghebaert, yeast cell walls do not completely disintegrate during cell milling [27]. In the present study, this could be a first reason as to why no asymptotic curve for the released protease activity is observed. It may be that this behavior is only observed with longer treatment times. In addition, the use of a vortex mixer instead of a flow through cell of commercial bead mills may have resulted in drastically lower local shear forces due to the lower power input into the system. On the other hand, it is also possible that the determined data point at 30 min cell disruption time is an outlier and that in fact, an asymptotic curve could be observed. Since this was not explored within the scope of this work, further experiments are required to identify the underlying mechanisms and trends. In particular, the use of a commercial cell mill is suggested for further investigations. For the scope of this study, it was decided to continue with 15 min cell disruption time for both ultrasound and glass bead treatment for better comparability.

**Figure 7:**
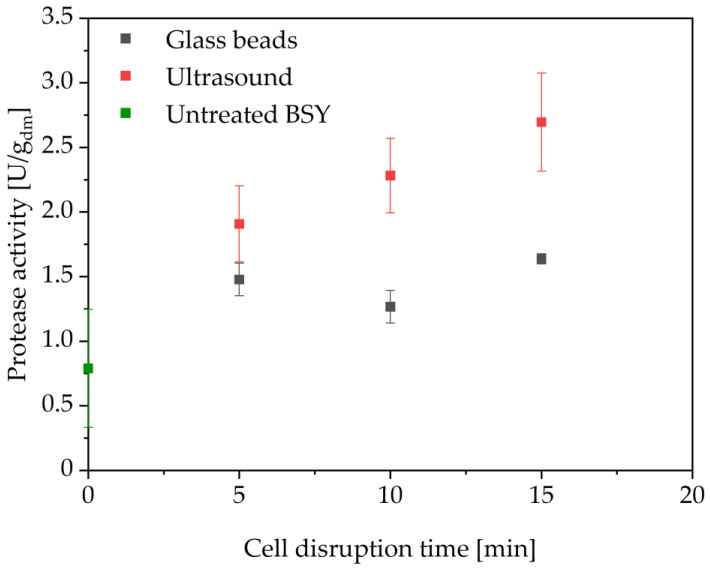
Protease activity in BSY extracts produced via 0.5 mm glass beads on a vortex mixer at 100% power input and 20 kHz ultrasound treatment. BSY suspended in 0.1 M sodium phosphate citrate buffer at pH 6. Differences between the two methods are significant at the 0.01 level using a two-tailed t-test. BSY: brewer’s spent yeast.

After the screening experiments were conducted, a fresh batch of BSY was produced, which was used for the autolysis, glass beads and ultrasound cell disruptions. The pH for all these disruptions was also adjusted to pH 6 according to the findings from the pH screening. The results for glass bead and ultrasound treatment are shown in Figure 7. All investigated cell disruptions resulted in proteolytic activity higher than for the untreated BSY supernatant, as can be seen in Figure 7. Ultrasound treatment resulted in higher protease activities than the glass bead treatment with a maximum activity of 2.70 ± 0.38 U/g_dm_ after 15 min, whereas glass bead treatment showed a better reproducibility, while only reaching a maximum activity of 1.64 ± 0.04 U/g_dm_ after 15 min. The poorer reproducibility of the ultrasound treatment might be due to its mechanism of cell disruption. This is based on asymmetrical bubble implosions via cavitation, which causes high shear-forces, disrupting the cell wall [52]. Depending on the positioning of the ultrasound probe in the sample, the location of these shear-forces will differ. Thus, slight deviations in the positioning of the probe in each of the triplicates might have led to differences in the effectiveness of the disruption process.

**Figure 8:**
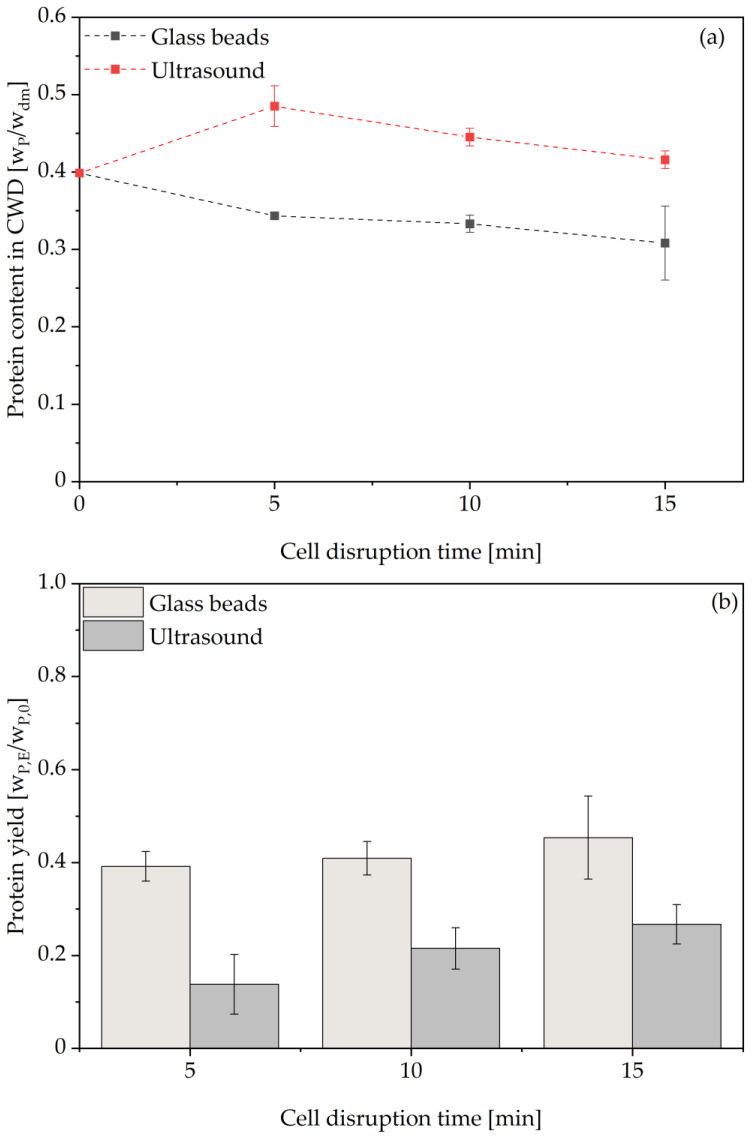
Protein content (**a**) in residual cell wall debris fraction of glass bead and ultrasound treated yeast cells at pH 6 in 0.1 M sodium phosphate citrate buffer. Protein extraction yield (**b**) for BSY extract calculated via mass balance. No significant differences between the two methods using a two-tailed t-test at a significance level of 0.05. CWD: cell wall debris.

The protein content in the residual cell wall debris for ultrasound treatment shows the same trend as for the autolysis samples with a dry mass specific increase from 39.9 ± 1.8% to 48.5 ± 2.6% followed by a linear decrease with progressing cell wall disruption to 41.6 ± 1.1%. However, as Figure 8a also shows, this behavior is different for the glass bead treatment. The protein content in the cell wall debris decreases for every investigated cell disruption time to a value of 30.8 ± 4.8%. This might be attributed to the mechanism of cell disruption, which is in itself unspecific. However, it could be shown that glass bead cell disruption can be selective towards releasing intracellular compounds, depending on the glass bead diameter. For yeast cells, glass beads with a diameter of 0.5–1.25 mm are commonly cited [45], where larger diameters are favored for releasing periplasmatic enzymes compared to smaller diameters being applied to release cytoplasmatic enzymes from *S. cerevisiae* [53]. This might also be true for other soluble intracellular compounds, which might not be extracted under the given cell disruption conditions, thus reducing the overall dry matter in the cell wall debris to a smaller extent. In order to test this hypothesis, measurements of carbohydrates and other intracellular components in both the BSY extracts and cell wall debris fractions would be required. Since these were not within the scope of the present work, the analysis is limited to protein extraction.

As can be seen in Figure 8b, protein extraction yields increased with increasing cell disruption time. Glass bead treatment was the more effective cell wall disruption method, resulting in a maximum protein extraction yield of 45.4 ± 8.9% by mass after 15 min compared to 26.7 ± 4.2% by mass released through ultrasound treatment. This is a considerably lower protein yield than that reported by other groups using these methods, especially when compared to Jacob et al., who stated 80% extraction effectiveness values after 15 min of glass bead and ultrasound treatment [25]. However, even though the same glass bead diameter and ratio to BSY suspension was used, there are differences in the experimental set-ups. A commercial cell mill was used by the other research groups, whereas a conventional vortex mixer had to be used in this study, presumably drastically lowering the effective shaking frequency and thus the protein extraction efficiency. Additionally, different yeast strains were used, the brewing process differed and no measure for the yeast vitality is given. It is therefore difficult to identify the main reason for these drastically different results.

**Figure 9:**
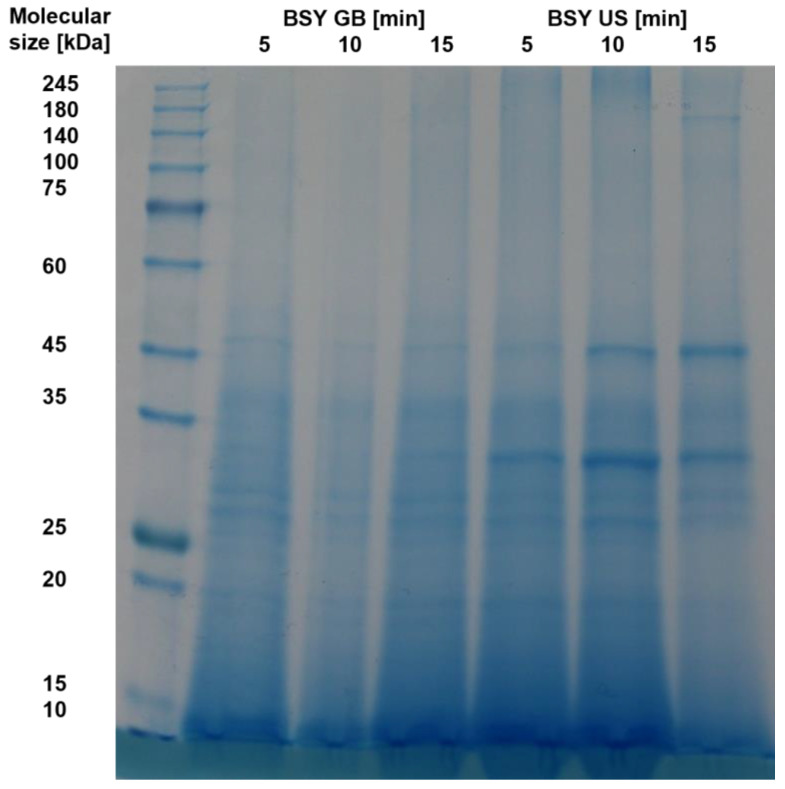
SDS-Page results for glass bead and ultrasound treated BSY extract samples. BSY: brewer’s spent yeast; GB: glass beads; US: ultrasound.

The qualitative analysis of released protein sizes via SDS-Page is shown in Figure 9 and resulted in similar results as the analysis for the thermal autolysis. Larger proteins above 45 kDa present after 5 min cell disruption seem to be degraded to smaller peptides. For glass bead treatment, the sample at 10 min cell disruption time presents the lightest blue color, indicating the lowest protein content. This is in line with the assayed protease activity at this time point, which was also the lowest of the three time points under investigation. A possible explanation might be that the protein release rate between 5–10 min was lower than the protein degradation rate due to the mechanical stress of the glass beads, meaning most of the present protein content was degraded to peptides below 15 kDa. This assumption is supported by the protein extraction yield, which increased in this time interval, while the protease activity decreased, possibly due to degradation of released proteases to non-functional peptides. The same assumptions apply for the ultrasound treatment, where an increase in color intensity in the range below 45 kDa is visible from 5 to 10 min before it is decreased again in the next 5 min interval.

### 3.4. High-Pressure Homogenization

High-pressure homogenization was chosen to evaluate a mechanical cell disruption method commonly used on an industrial scale [45]. Since the production capacity per brew in the microbrewery Campus Perle is limited to 1.5–2 L of BSY suspension, and the homogenizer has a feed volume of 300 mL per run, a second beer fermentation was started. Table 3 summarizes the differences in fermentation time until a constant residual extract of 3.5 °P was reached, as well as the differences in yeast vitality after harvest. The intrinsic ability to metabolize glycogen of the second BSY batch, represented by the AP_10_ value, was lower than that of the first batch. However, the ability to metabolize an exogenous glucose substrate, represented by the AP_20_ value, was greater. Further studies not included in this paper have shown that both AP_10_ and AP_20_ are positively and statistically significantly correlated with residual protease activity based on Pearson r and a two-tailed t-test. However, the AP_20_ value has a stronger positive correlation with protease activity (Pearson r of 0.92 compared to 0.77). Additionally, by determining an intracellular pH (ICP) as a second measure of yeast vitality for subsequent brews, it was shown that only the AP_20_ value positively and statistically significantly correlated with the ICP (data included in Supplementary Materials Tables S1 and S2). In summary, a higher AP_20_ as a measure of BSY vitality indicates a higher proteolytic activity for BSY extracts, whereas the AP_10_ value is not suitable to derive clear trends for this parameter.

**Figure 10:**
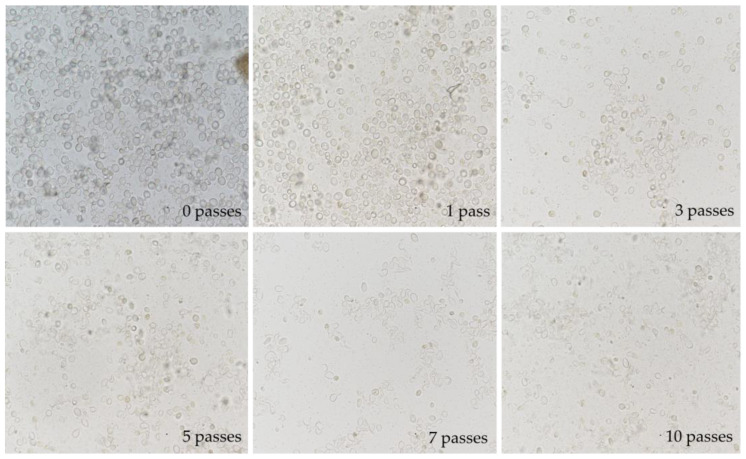
Image-based analysis of cell disruption progress with increasing number of passes through high pressure homogenization at 600 bar. Flux of 9 l/h maintained at max. 20 °C with countercurrent tubular heat exchanger at outlet of HPH valve. Top left to bottom right: 0, 1, 3, 5, 7 and 10 passes through homogenization valve.

Before investigating the suitability of high-pressure homogenization for the preparation of proteolytic BSY extracts, the general suitability of this cell disruption method was analyzed by image-based analysis. Figure 10 summarizes the results obtained for cell disruption at 600 bar for 0 to 10 passes through the homogenization chamber.

It can be clearly seen that the degree of cell disruption increases as the number of passes increases. The cell wall is effectively disrupted using this technique. The images were digitally analyzed for the degree of cell disruption. The results are summarized in Table 4. For all investigated pressure levels, a grade of cell disruption above 80% was achieved after 10 passes. Higher homogenization pressures yielded higher degrees of cell disruption at a lower number of passes. Depending on the application of the final product, optimum conditions for cell disruption need to be identified, also taking into account processing times and energy requirements.

**Figure 11:**
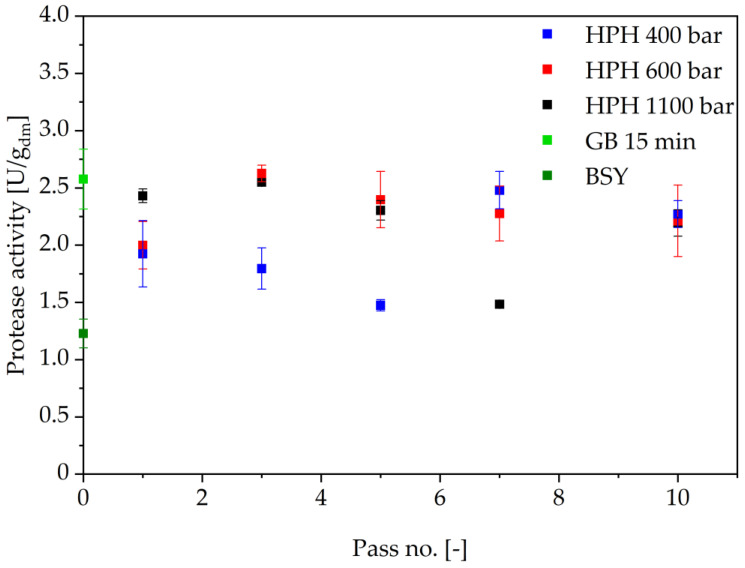
Protease activity in high pressure homogenized BSY extracts. Significant differences in a two-tailed t-test between runs at 600 bar and 1100 bar at a significant level of 0.01. No significant differences between all other combinations. HPH: high pressure homogenization; GB: glass beads; BSY: brewer’s spent yeast.

There is no clear trend in the release of proteolytic activity as a function of pressure and number of passes, as shown in Figure 11. All tested homogenization pressures and pass numbers led to an increased proteolytic activity in the BSY extract compared to the negative control of non-homogenized BSY. Since the experiment was conducted with a different batch of BSY than the previous cell disruption methods, a glass bead cell disruption for 15 min at 4 °C using 0.5 mm glass beads in 0.1 M sodium phosphate citrate buffer at pH 6 was conducted. The released protease activity in the BSY extract from glass bead treatment with 2.6 ± 0.3 U/g_dm_ is in the same order of magnitude as that of the extracts produced through HPH pressures of 600 and 1000 bar and one to three passes. Generally, HPH pressures above 400 bar seem to release protease activity more effectively at pass numbers below seven. This might be due to the already progressed disintegration of the cell wall through the high dynamic pressure differences and the impingement in the exit zone of the valve [54]. However, the overall maximum released proteolytic activity after one pass at 1100 bar is not considerably increased with further passes, opening the potential to introduce a continuous processing route through the homogenizer. This behavior might occur due to the shear undirected force during HPH, leading to degradation of the released proteases from the previous pass with every next pass through the homogenization chamber.

**Figure 12:**
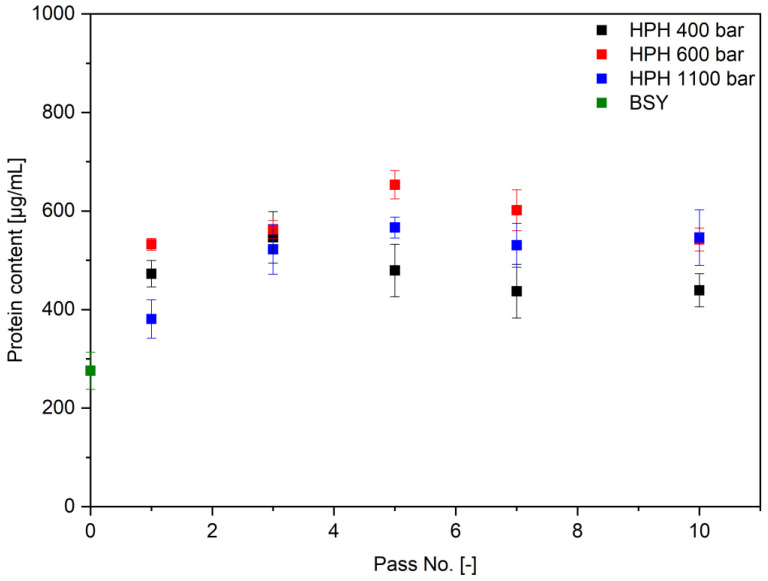
Pierce protein content in high pressure homogenized BSY extracts at pH 6 in 0.1 M sodium phosphate citrate buffer. Differences between all pairs of homogenization pressures are significant at the 0.01 level using a two-tailed t-test. HPH: high pressure homogenization; BSY: brewer’s spent yeast.

**Figure 13:**
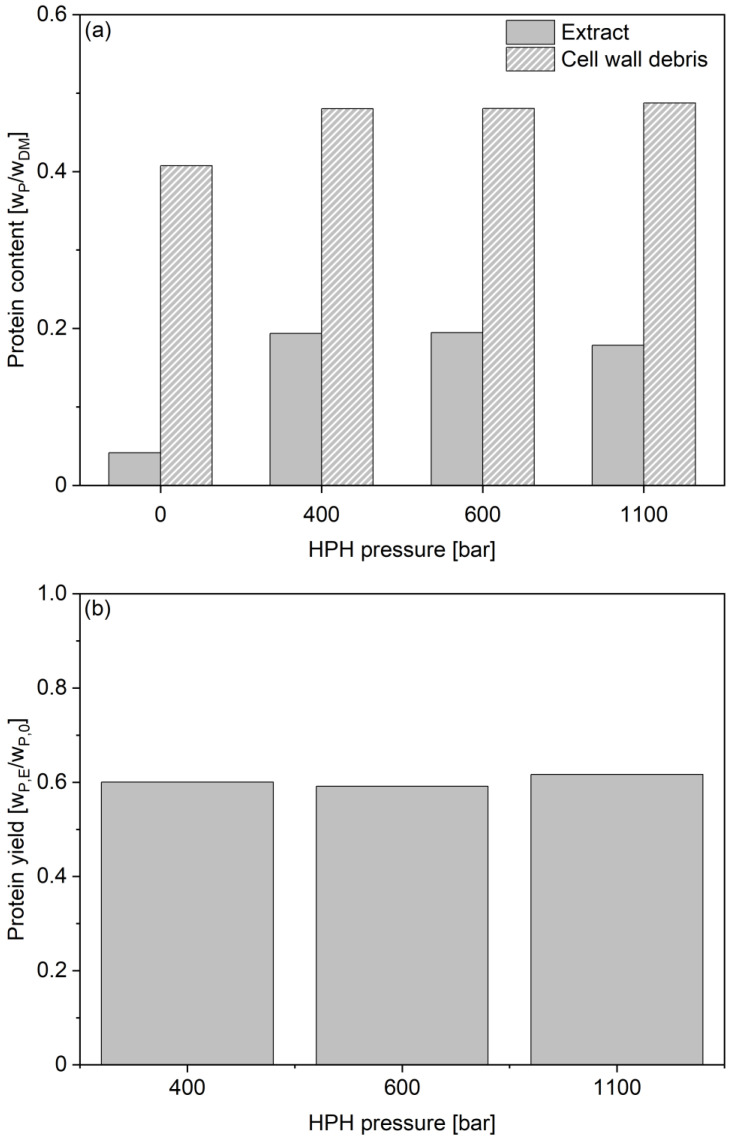
Protein content (**a**) in BSY extract and residual cell wall debris fraction of high-pressure homogenized yeast cells at pH 6 in 0.1 M sodium phosphate citrate buffer determined via amino acid HPLC. Protein extraction yields (**b**) for BSY extracts calculated via mass balance. All protein data for a total pass number of 10. No statistical evaluation possible due to single technical replicate runs. HPH: high-pressure homogenization.

Next to proteolytic activity, a common parameter to evaluate cell disruption efficiency is the overall protein extraction efficiency. Figure 12 summarizes the obtained protein contents in the BSY extracts determined via a Pierce assay. The released protein content increases until three passes for 400 bar and until five passes for 600 and 1100 bar, before decreasing again. The apparent decrease in the protein content might be counterintuitive with the progressing cell disruption as visible by the image-based analysis. However, it is hypothesized that the maximum extractable, non-cell wall bound protein content has already been released after these pass numbers. The result would be a fragmentation of the extracted proteins to smaller peptide fractions with further passes through the homogenization valve. Since the Pierce assay has a lower detection limit for peptides, the apparent decrease in protein content in the supernatant might be attributed to the generation of peptides smaller than this, as Jacob et al. also reported for the investigated BSY autolysates analyzed via Bradford assay [38]. This assumption is further supported by the amino acid HPLC results presented in Figure 13a for the total protein contents in the extracts and cell wall debris fractions after 10 passes. All investigated pressure levels resulted in protein contents of 19% for the BSY extracts and 48% for the residual cell wall debris fractions.

Additionally, the overall protein extraction yields of 60%, shown in Figure 13b, were reached for all investigated pressure levels after 10 passes. For 400 bar, this protein yield has already been found after five passes through the homogenization valve, further supporting the assumption that the cell wall disruption at all investigated pressures has already effectively released all non-cell wall bound protein. However, due to the sample mass required for HPLC analysis, the measurement for the protein content and resulting yields was only a single measurement each, eliminating the possibility of analyzing statistical relevance of the results. Achieving protein extraction yields of 60% by mass for yeast is higher than that reported in previous studies. Lee et al. reported protein extraction efficiencies of <20% for 600 bar homogenization pressure and <40% for 1200 bar homogenization pressure for instant dried yeast suspensions of *S. cerevisiae* of 2.3 × 10^8^ CFU/mL [55]. However, a different homogenizer of the type NLM 100 by Ilsin Autoclave Co., Ltd. was used in their study. As Moore et al. found using a single piston positive displacement pump APV Gaulin 15 M coupled to an APV Junior plate heat exchanger by APV Baker, the efficiency of cell disruption of yeasts in a high pressure homogenizer strongly depends on the valve geometry and impact ring dimensions, which might explain the reported lower extraction yields [54]. Further, on a similar homogenizer model type used by Verduyn et al., namely a Panda Homogenizer by GEA Niro-Soavi, lower protein recoveries were reported. For a homogenization of *S. cerevisiae* grown on sugar cane molasses, a protein recovery of >45% after homogenization at 1000 bar for three passes and of >25% after homogenization at 600 bar for six passes was found [56]. It has to be mentioned that all cited publications determined the protein contents via colorimetric assays or via Kjeldahl measurements using a nitrogen conversion factor of 6.25. Even though Verduyn et al. corrected for free ammonia contents, these indirect types of protein content determination are prone to overestimate protein contents [57,58]. The commonly used conversion factor of 6.25 as a ratio to convert total nitrogen into protein contents proposed by Jones in 1930 was originally intended to be used for food and feed [59]. For yeasts, a nitrogen conversion factor of 5.5 was proposed by Reed et al. in 1990 to account for non-proteinogenic nitrogen such as nucleic acids or free ammonia [60].

**Figure 14:**
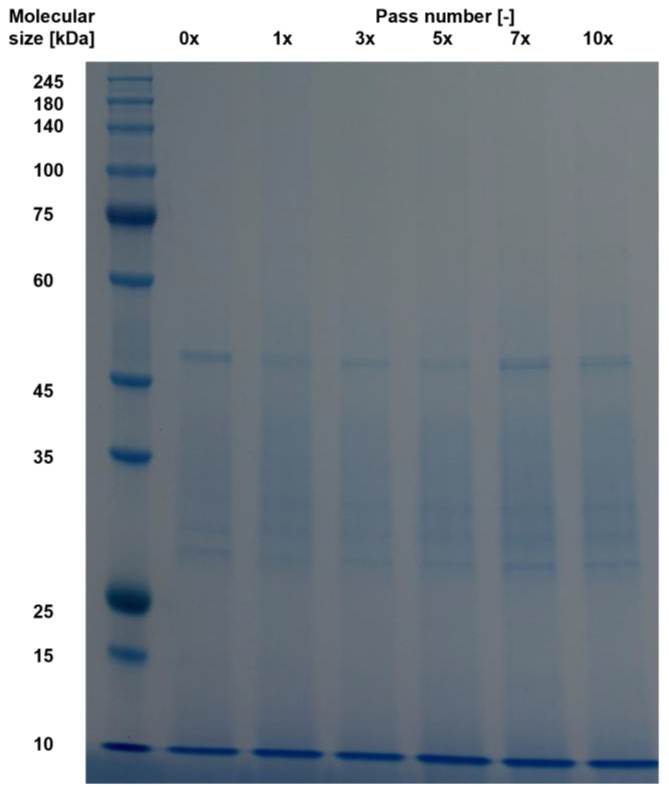
SDS-Page results for 600 bar high pressure homogenized BSY extract samples.

The conducted SDS-Page is given in Figure 14 and shows that no clear protein bands above 45–50 kDa are present. Furthermore, all protein bands below this size intensify in color with an increasing pass number, which could be related to the breakdown of proteins extracted in the previous pass through the homogenization chamber to smaller peptides through the high mechanical stress applied. The prominent bands are in line with previously reported protein sizes extracted via SDS-Page from wine yeast *S. cerevisiae* [61].

### 3.5. Overall Comparison of Cell Disruption Methods

**Figure 15:**
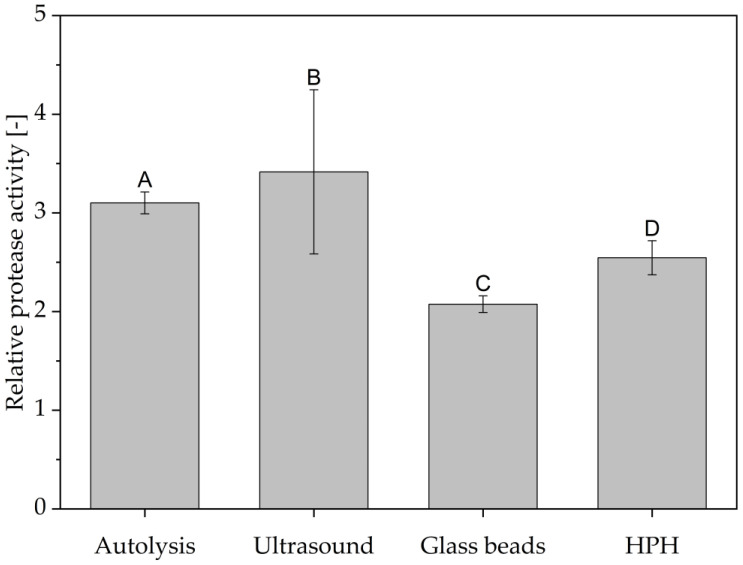
Comparison of highest determined protease activity per cell disruption method. Activity reported as relative activity to the BSY blank activity of 0.95 ± 0.03 U/g_dm_ for HPH and 0.79 ± 0.46 U/g_dm_ for all other methods. Autolysis at 45 °C for 3 h. Ultrasound and glass bead treatment for 15 min. HPH at 600 bar and three passes. Differences between the groups are significant at a *p*-value of <0.01 according to a one-way ANOVA, indicated by the capital letters A, B, C and D, each of them corresponding to a group. HPH: high-pressure homogenization.

In addition to an evaluation of the released protease activities and protein yields for the individual methods, a comparison of the methods to each other is also of interest. To enable this comparison for the released protease activities, a relative activity is defined and given in Figure 15. This relative activity is based on the ratio of released proteolytic activity in the produced extract to the BSY blank activity for each brew. Only the highest determined activity per method has been used. It has been demonstrated that autolysis and ultrasound treatment result in extracts exhibiting comparable relative protease activity, with values of 3.10 ± 0.11 and 3.42 ± 0.83, respectively. Autolysis is recognized as a process that releases a significant amount of protease enzymes, a conclusion that has been reached by several research groups, and evidenced by the presence of high peptide and free amino acid contents [25,26,44]. The results of this study lend support to this hypothesis, insofar as autolysis was shown to release the highest protease activity among the four cell disruption methods that were investigated. However, due to the limited number of sampling times, it remains unclear whether the protease activity that was determined is in fact the highest possible activity released during the cell disruption process. This leads to an additional challenge in the scope of an application of autolysis for the production of proteolytic extracts. The influence of various factors, including the brewing style, yeast vitality, the storage period prior to cell disruption and the selected buffer type, on the resulting yeast metabolism at the onset of autolysis results in the variability of the time of highest protease activity between batches. The absence of an in-line enzyme activity assay limits the capacity of automated process control, while offline analysis is too slow to serve as a stopping criterion.

**Figure 16:**
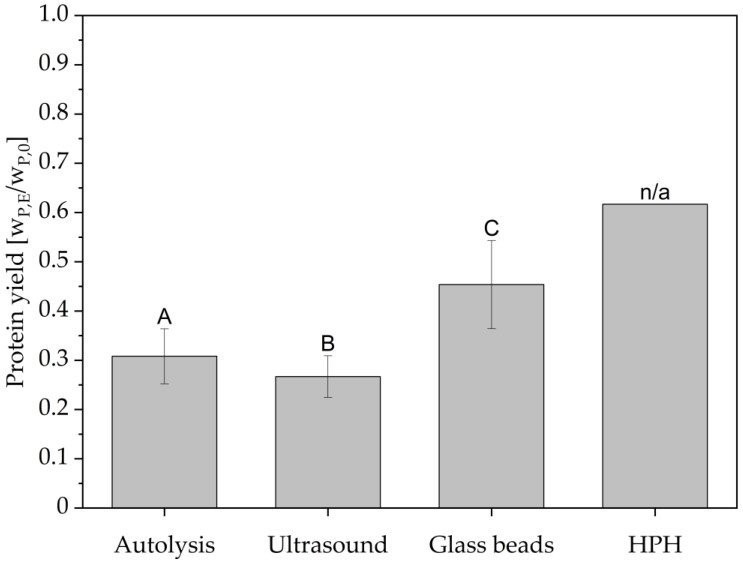
Comparison of highest determined protein yield per cell disruption method. Autolysis at 50 °C for 4 h. Ultrasound and glass bead treatment for 15 min. HPH at 1100 bar and 10 passes. Differences between the groups are significant at a *p*-value of <0.01 according to a one-way ANOVA, indicated by the capital letters A, B and C, each of them corresponding to a group. HPH: high-pressure homogenization.

Ultrasound treatment, on the other hand, showed an asymptotic trend towards released protease activity in the extract. With data available for the yeast strain and brewing process in question, a first order type kinetic could be set up to predict the potential time point of highest protease activity. This approach could lead to similar results than already reported by multiple different research groups for protein release for yeast [25,46,47]. Potential challenges with ultrasound treatment lie in its apparently low reproducibility, as can be exemplified in Figure 15. The poor reproducibility of the treatment may be attributed to the positioning of the ultrasound probe in the BSY suspension, which affects the efficiency of cell disruption. Local effects of bubble cavitation and associated high temperature gradients may result in non-uniform protease inactivation. In contrast, Jacob et al. did not report comparably poorer reproducibility when investigating the effect of cell disruption method on released protein content from BSY [25]. This is also the case in the present study for protein content, as can be seen in Figure 16, which might indicate that this technology is better applied in the field of protein extraction than for retrieving intrinsic bioactivities. Additional limitations for ultrasound treatment occur in the field of upscaling, where penetration depth of the amplitude and overall energy input have been reported to be challenging [26]. However, research groups like Bystryak et al. aimed at overcoming these limitations by introducing a scalable high-intensity ultrasound system [52].

The application of high-pressure homogenization resulted in a decrease in relative protease activity in the extracts when compared with autolysis and ultrasound treatment, with a value of 2.55 ± 0.17. However, this technology is the most widely used cell disruption method on an industrial level, thus showing potential for rapid adoption of the proposed production of proteolytic extracts. [26,52]. Since there was no clear trend for protease activity with increasing pass number, this technology has the potential to be run continuously, applying only one pass. In addition, this technology could be relevant for extracting protein from BSY, as this cell disruption method led to the highest overall protein yield in the present study, with 61.7% at 1100 bar after 10 passes, as shown in Figure 16. However, the aim of this study was to maximize the extracted protease activity in the BSY extracts, with protein yield being only secondary information. For protein extraction from BSY, extensive studies already exist that highlight the potential of e.g., thermal yeast autolysis, to result in protein extraction yields close to 100% when run for ≥24 h [25,40].

Glass bead treatment resulted in the lowest relative protease activity with 2.07 ± 0.08. However, since cell mills have been used on an industrial scale for decades in the disruption of yeast cells to retrieve intracellular components, this cell disruption method should be investigated in more detail [26,62]. In the present study, no commercial cell mill was available for testing, which led to the use of a vortex mixer instead. The comparability between the obtained results with a commercial bead mill are unknown and thus have to be tested experimentally to show its potential for commercial use.

The overall comparison of qualitative protein size analysis revealed that, for all four investigated cell disruption methods, a decrease in protein and peptide size can be observed with increasing cell disruption time in the case of thermal autolysis, glass bead and ultrasound treatment and pass number in the case of high-pressure homogenization. For the three mechanical cell disruption methods, this is hypothesized to be due to the undirected forces of cell disruption, also acting on already extracted proteins in the extracellular medium, thus reducing their size to form peptides over time or pass number, respectively. For thermal yeast autolysis, the main reason for an increasing peptide and free amino acid content is hypothesized to be due to the action of (exo-)peptidases.

## 4. Conclusions

All four investigated cell disruption methods, namely thermal autolysis, ultrasonication, glass bead treatment and high-pressure homogenization, resulted in proteolytically active BSY extracts. It was shown that autolysis and ultrasound treatment lead to extracts with the same order of magnitude for released protease activity. However, due to challenges in process control regarding a missing inline determination of protease activity for stopping the thermal yeast autolysis at the point of highest activity, this method is deemed unsuitable for the production of proteolytically active BSY extracts. Current technical advances in ultrasound treatments try to overcome known scalability challenges with this technique [52]. However, due to ease of scalability, broad application in the industry and the shown potential for continuous operation by just applying one pass at 1100 bar pressure through the homogenizer, this cell disruption technique shows great potential for application in the production of BSY extracts. In the present study, HPH also resulted in the highest overall protein yield with 60% after five passes at 400 bar homogenization pressure. However, since thermal yeast autolysis was stopped after 4 h in the scope of this study, this technique should not be disregarded for the production of amino acid and peptide-rich BSY extracts. Other studies have reported protein yields >60% for BSY when run for 24–48 h, leading to non-enzymatic bioactivities in the produced yeast extracts [25,40,63]. Further investigations are thus required to assess techno-economic factors for the desired products, while taking product specific energy requirements into account. Further works should also assess the scalability and reproducibility of the obtained results for high-pressure homogenization, especially focusing on brewing-type influences such as number of re-pitching cycles of the yeast prior to harvest. Emphasis should be placed on a reproducible method to determine the yeast vitality, such as flow cytometry methods for accurate intracellular pH determination proposed by Weigert et al. and Eigenfeld et al. [64,65]. Assessing the metabolic state of the yeast cells prior to cell disruption might enable a prediction of the order of magnitude of the resulting proteolytic activity in the yeast extracts.

## Figures and Tables

**Table 1:** List of buffers used during pH screening experiments.

pH [-]	Buffer [0.1 M]
3–7	Na_2_HPO_4_ + C_6_H_8_O_7_
8–9	C_4_H_11_NO_3_ + HCl
10	Na_2_CO_3_ + NaHCO_3_

**Table 2:** Known vacuolar proteases in *S. cerevisiae* and corresponding molecular sizes [34,43].

Protease	Molecular Size [kDa]
Dipeptidylaminopeptidase B	90–120
Carboxypeptidase S	65–75
Aminopeptidase Y	60–75
Carboxypeptidase Y	60
Aminopeptidase I	50–57
Proteinase A	42–45
Proteinase B	31–37

**Table 3:** Summary of fermentation, vitality and protease activity differences for the two BSY batches. Protease activity determined after 15 min glass bead treatment at 4 °C using 0.5 mm glass beads on a vortex mixer. BSY: brewer’s spent yeast; AP_10_: magnitude of spontaneous acidification; AP_20_: magnitude of glucose induced acidification power; v_S_: mass specific activity; GB: glass beads; US: ultrasound; HPH: high-pressure homogenization.

BSY Batch Designation	Fermentation Time [d]	Cold Storage Time [d]	AP_10_ [-]	AP_20_ [-]	v_S_ [U/g_dm_]
Autolysis, GB, US	11	3	1.51 ± 0.01	1.85 ± 0.01	1.64 ± 0.04
HPH	9	2	1.36 ± 0.01	2.15 ± 0.01	2.58 ± 0.26

**Table 4:** Grade of cell disruption from image-based analysis of high pressure homogenized BSY samples.

Homogenization Pressure [Bar]	Pass Number [-]	Grade of Cell Disruption [%]
400	1	50
	3	60
	5	65
	7	75
	10	80
600	1	50
	3	75
	5	80
	7	90
	10	>95
1100	1	75
	3	80
	5	>95
	7	>95
	10	>98

## Data Availability

The original contributions presented in the study are included in the article and Supplementary Materials. Further inquiries can be directed to the corresponding authors.

## Supplementary Materials

The following supporting information can be downloaded at: https://www.mdpi.com/article/10.3390/foods14030503/s1, Figure S1: Proteolytic activity of thermally autolyzed yeast extracts over 24 h runs in 0.1 M sodium phosphate citrate buffer at pH 7.; Table S1: Yeast vitality data for different brews. Cell disruption via 15 min glass bead treatment in 0.1 M sodium phosphate citrate buffer at pH 6; Table S2: Correlation and significance analysis of yeast vitality and protease activity. Significance level of 0.05 and two-tailed t-test used.
