# Supplementary material for: From Waste to Value: Extraction of Protease Enzymes from Brewer’s Spent Yeast
Source: Foods. 2025 Feb 5;14(3):503. doi: 10.3390/foods14030503 (PMC11817683; doi:10.3390/foods14030503)
Supplement: Supplementary file 1 [file foods-14-00503-s001.zip › foods-3405495-supplementary.pdf]

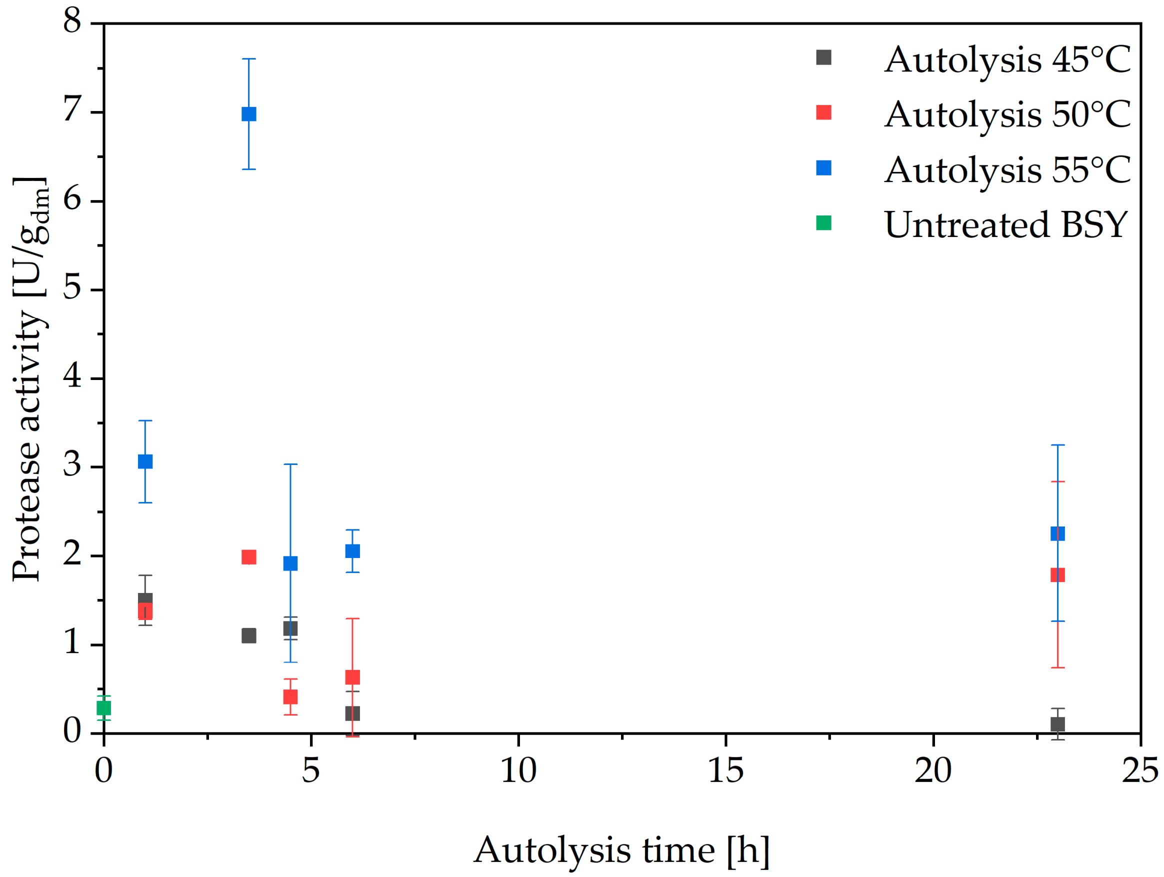

Figure S1: Proteolytic activity of thermally autolyzed yeast extracts over 24 h runs in 0.1 M sodium phosphate citrate buffer at pH 7.

Table S1: Yeast vitality data for different brews. Cell disruption via 15 min glass bead treatment in 0.1 M sodium phosphate citrate buffer at pH 6.

Yeast strain	$t_{Fermentation}$ [d]	$t_{ColdStorage}$ [d]	AP_{10} [-]	AP_{20} [-]	ICP [-]	$a_{Protease}$ [U/g_{DM}]
S. cerevisiae W-34/70	13	1	1.8	2.32	-	5.95
S. cerevisiae W-34/70	11	1	1.81	2.33	-	4.63
S. cerevisiae W-34/70	9	2	1.37	2.16	-	2.58
S. cerevisiae W-34/70	11	3	1.3	2.2	6.89	4.03
S. cerevisiae W-34/71	11	3	1.51	1.85	6.49	1.67
S. cerevisiae W-34/70	13	1	1.41	2.02	6.69	2.80
Bottom-fermenting lager yeast	Unkown	1	0.97	1.89	6.60	1.15

Table S2: Correlation and significance analysis of yeast vitality and protease activity. Significance level of 0.05 and two-tailed t-test used.

Parameter	$r_{Pearson}$	t-value	Degrees of freedom	t_{crit}	$t > t_{crit}$
AP_{10} + $a_{Protease}$	0.771	9.53	5	2.57	6.96
AP_{20} + $a_{Protease}$	0.917	28.66	5	2.57	26.09
ICP + $a_{Protease}$	0.900	9.52	5	2.57	6.95
AP_{10} + ICP	-0.105	-0.53	2	4.30	-4.83
AP_{20} + ICP	0.986	176.39	2	4.30	172.09
